# *Leishmania tarentolae*: an alternative approach to the production of monoclonal antibodies to treat emerging viral infections

**DOI:** 10.1186/2049-9957-4-8

**Published:** 2015-04-01

**Authors:** Joshua D Jones

**Affiliations:** Division of Virology, Department of Pathology, University of Cambridge, Tennis Court Road, Cambridge, CB2 1QP UK

**Keywords:** Antibody, Ebola, Emerging, Expression, Infection, *Leishmania*, Monoclonal, Therapy

## Abstract

**Background:**

Monoclonal antibody therapy has an important role to play as a post-exposure prophylactic and therapeutic for the treatment of viral infections, including emerging infections. For example, several patients of the present Ebola virus outbreak in West Africa were treated with ZMapp, a cocktail of three monoclonal antibodies which are expressed in *Nicotiana benthamiana*.

**Discussion:**

The majority of monoclonal antibodies in clinical use are expressed in mammalian cell lines which offer native folding and glycosylation of the expressed antibody. Monoclonal antibody expression in vegetal systems offers advantages over expression in mammalian cell lines, including improved potential for scale up and reduced costs. In this paper, I highlight the advantages of an upcoming protozoal system for the expression of recombinant antibody formats. *Leishmania tarentolae* offers a robust, economical expression of proteins with mammalian glycosylation patterns expressed in stable cell lines and grown in suspension culture. Several advantages of this system make it particularly suited for use in developing contexts.

**Summary:**

Given the potential importance of monoclonal antibody therapy in the containment of emerging viral infections, novel and alternative strategies to improve production must be explored.

**Electronic supplementary material:**

The online version of this article (doi:10.1186/2049-9957-4-8) contains supplementary material, which is available to authorized users.

## Multilingual abstracts

Please see Additional file 1 for translations of the abstract into the six official working languages of the United Nations.

## Background

Monoclonal antibodies (mAbs) are used in wide range of contexts, including biomedical research and in the diagnosis and treatment of diseases. In recent decades, much research has been conducted into the development of mAbs as therapeutics for cancer [1], autoimmune conditions [2] and infectious diseases [3]. The majority of mAbs in clinical use are human, chimeric or humanised [4], and these molecules retain the native antibody structure, containing both the fragment antigen-binding (Fab) and fragment crystallisable (Fc) domains [5]. The Fc domain determines the range of biological effects an antibody isotype may have. For example, the antibodies of the IgG1 isotype are better opsonins than those of the IgG4 isotype [6]. The advantages of mAb therapy include low toxicity, high specificity and versatility, with the range of biological effects dependent upon the Fc region. Functions of mAbs reflect the functions of native antibody isotypes and can include pathogen opsonisation, complement activation, antibody-dependent cell cytotoxicity and virus neutralisation.

**Figure 1:**
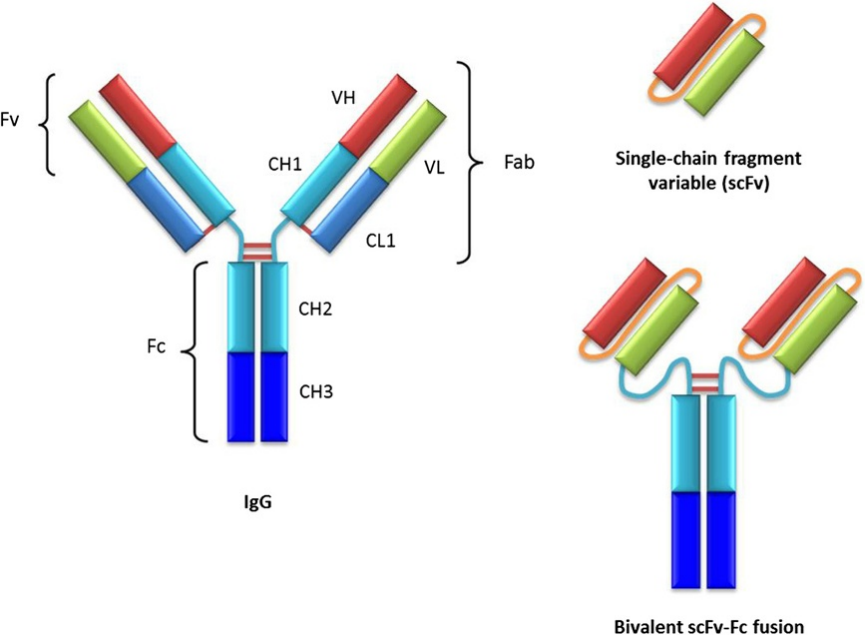
The structures of native IgG, scFv and bivalent scFv-Fc fusion molecules. Red and green: variable domains; blue: constant domains; brown: disulphide bonds; orange: artificial peptide linker.

In recent years, however, many new recombinant antibody fragment formats have been developed which lack the Fc domain [7]. These include single-chain fragment variable molecules (scFvs) in which a flexible amino acid linker joins two variable domains, as illustrated in Figure 1[8]. Instead of using the biological functions imparted by the Fc domain, antibody fragment formats are used when only antigen binding is required, for example in diagnostic assays, drug delivery or virus neutralisation [9, 10]. A recombinant bivalent antibody format analogous to the native antibody can be produced by the fusion of an scFv to an Fc domain (see Figure 1) [11]. These recombinant bivalent molecules have improved affinity and serum half-life relative to scFvs [12], and have demonstrated efficacy in mice against Venezuelan equine encephalitis virus [13].

Antibody therapy as a treatment for infectious disease has its origins in the ‘serum therapy’ treatments of the 1890s, and today mAbs are licensed for passive antibody therapy to treat a range of viral infections including respiratory syncytial virus, hepatitis A, hepatitis B and varicella-zoster virus (reviewed in [14]). Neutralising mAbs have an important role to play in the treatment of emerging infections for which vaccines are presently unavailable. For example, in the 2010 outbreak of Hendra virus in Queensland, Australia, two patients were treated with neutralising mAbs specific for the henipavirus G envelope glycoprotein receptor binding domain [15, 16]. More recently, a cocktail of neutralising mAbs specific for Ebola virus (EBOV), ZMapp, has shown promising results in non-human primates (NHPs) [17], and was used in the treatment of several patients of the current EBOV outbreak in West Africa [18]. Therefore, where vaccination is immunologically challenging to develop, or an efficacious vaccine is yet to be produced, as in the case of EBOV, mAbs offer an invaluable therapeutic alternative. This article will discuss the importance of considering a sustainable expression system for mAbs and will highlight a protozoal expression system which may be particularly suited to use in developing contexts.

## Discussion

At the time of writing (December 2014), the total number of cases recorded in the current EBOV outbreak in West Africa has reached an estimated 18,000, with some 6,400 deaths. Given the severity of the outbreak, a small number of patients were treated with ZMapp, a cocktail of three mAbs, but the limited supply of ZMapp was soon exhausted [18]. Although only given to a small number of patients, the efficacy of ZMapp has been demonstrated in NHPs, and the antibodies which comprise the cocktail are presently expressed in *Nicotiana benthamiana*[17]. While further safety and efficacy testing of ZMapp is required before it is routinely used to treat EBOV, the nature of the expression vector must also be considered. Foremost amongst the requirements of a system suitable for the expression of mAbs are yield, cost effectiveness, potential for scale up, ease of purification, low risk of hazardous viral contamination and native folding and glycosylation of the expressed antibody [11, 19]. Differences between the glycosylation profiles of administered mAbs and the cells of the recipient are known to increase the immunogenicity of the expressed mAb [20, 21]. Moreover, subtle alterations in the glycosylation of the Fc region are known to alter the antibody effector functions [22].

The requirement for a native glycosylation profile to minimise potential immunogenicity remains a key reason why the majority of currently approved therapeutic mAbs are expressed in mammalian cell lines, despite relatively high production costs which are not significantly mitigated by scaling up [11]. However, the potential immunogenicity of mAbs expressed in *N. benthamiana* has been addressed by the use of ribonucleic acid (RNA) interference technology to engineer plants with a humanised glycosylation profile [23]. Research has also been undertaken into the humanising of well-characterised bacterial expression systems. For example, the recent transfer of a *Campylobacter* N-linked glycosylation cassette into *Escherichia coli* raises the possibility of the humanisation of bacterial expression systems [24]. However, bacterial expression systems are typically unsuitable for native mAb expression as they lack both the sophisticated folding apparatus and post-translational modifications required for native mAb production. Instead, bacterial systems have been widely used for the expression of a variety of recombinant antibody formats, including Fab and scFvs [11]. Importantly, recent research has suggested that a protozoal expression system may be able to express recombinant antibody formats with a mammalian glycosylation pattern.

The non-pathogenic eukaryotic protozoan *Leishmania tarentolae*, whose genome was sequenced in 2012 [25], has been used to investigate RNA editing [26], gene amplification [27] and as a vaccine candidate for the disease leishmaniasis [28]. Since its description as an expression system in 2002 [29], *L. tarentolae* has found a range of applications, including the expression of human soluble amyloid precursor protein alpha, a cleavage product of amyloid precursor protein, the aetiological agent of Alzheimer’s disease [30]. The advantages of the *L. tarentolae* expression system include facile handling of cultures, an optimal culture temperature of 26°C, rapid growth rate (with a doubling time of approximately six hours in agitated culture), high cell densities (up to 10^9^ cells/ml), promising potential for scale up, the ability to produce stable cell lines and a complete eukaryotic folding and post-translational modification machinery, including a mammalian glycosylation profile [29, 31–33]. Recent research has demonstrated the use of *L. tarentolae* to express scFvs fused to a rabbit Fc region, which resulted in a recombinant bivalent antibody format similar to the native antibody structure [34]. The authors also demonstrated the ease of purification of the secreted scFv-Fc fusions from the culture media using one-step affinity purification. As mentioned above, scFv-Fc fusions have enhanced affinity, a reduced propensity to aggregate and significant improvements in serum half-life relative to scFvs [12]. Therefore, *L. tarentolae* represents a eukaryotic expression platform with the potential to produce high yields of neutralising scFv-Fc fusion proteins.

As we have seen with the present EBOV outbreak in West Africa, neutralising mAbs have the potential to play a key role in the containment of emerging viral infections. In the absence of a vaccine, mAbs can be effectively employed as a post-exposure prophylactic, while mAbs may continue to be used alongside a vaccine as a post-exposure therapeutic. Therefore, there is a need for cheap and scalable expression systems which can be rapidly employed to express high yields of recombinant antibody formats. The majority of mAbs in clinical use are expressed in mammalian cell lines, typically Chinese hamster ovary cells. The advantages of expression in such mammalian cell lines include native post-translational modifications, the production of stable cell lines and large- scale suspension culture [35]. Recombinant mAb expression in *N. benthamiana* offers advantages over mammalian cell lines, principally significantly lower costs, more efficient potential for scale up and, in engineered plants, a more uniform humanised glycosylation profile [11, 22]. However, the *L. tarentolae* expression platform must be developed alongside these systems as a potential alternative for the expression of neutralising mAbs, including ZMapp. This platform offers a robust and cost-effective alternative, with the potential for high yields of easily purified recombinant antibody formats with mammalian glycosylation patterns expressed in stable cell lines grown in suspension. If we are to envisage mAbs as a key part of the containment of emerging viral infections, we must interrogate the best strategies to rapidly and cheaply produce sufficient quantities.

Moreover, several features of *L. tarentolae* make it particularly suited to recombinant antibody production in developing contexts; in particular, the ease of handling, low production costs and ease of purification. As a tropical protozoan with an optimal growth temperature of 26°C, the potential for *L. tarentolae* to be cultured at ambient temperature in tropical developing contexts should be evaluated, as ambient culture would significantly reduce production costs. The potential for ambient culture may be further enhanced by the development of strains with greater tolerance for a range of temperatures to reflect the variation in daily temperatures. While the majority of industrial recombinant protein expression occurs in developed nations, in the long term, the involvement of developing nations in the production of mAbs could bring many benefits to the containment of emerging infections and the nations involved. However, I acknowledge that there are potential legal and regulatory obstacles to the production of mAbs in developing contexts, including issues of intellectual property and regulation. Ultimately, the demand in developed countries for mAb therapy for the treatment of conditions including cancer and autoimmunity will continue to rise, making the pursuit of alternative, more economical mAb expression systems likely to benefit pharmaceutical companies marketing their products in developed contexts. The production of mAbs in developing contexts would bring associated capacity building that would also deliver much-needed jobs and education. Moreover, the capacity to rapidly produce and purify mAbs at the frontline of emerging viral infections could significantly aid containment, and reduce both the cold chain to affected regions and logistical obstacles to the dissemination of treatment.

## Summary

In this paper, I have used the recent EBOV outbreak in West Africa to illustrate the need to continue to develop alternative, sustainable, strategies for mAb expression. Platforms for mAb expression must offer sustainable, rapid, cost-effective yields of easily purified recombinant mAb formats with a mammalian glycosylation pattern [11, 19]. The majority of mAbs in clinical use are expressed in mammalian cell lines, which entail high production costs not significantly mitigated by scale up. Here I have sought to highlight the potential value of the developing *L. tarentolae* expression platform. In particular, the recent expression of an scFv-Fc fusion in *L. tarentolae* raises the possibility that this platform could offer sustainable, cost-effective, production of recombinant mAb formats with mammalian a glycosylation pattern [34]. The refinement of strategies for rapid and sustainable expression of mAbs must consider *L. tarentolae* alongside strategies already in use, such as *N. benthamiana*. Moreover, the *L. tarentolae* system is suited to use in developing contexts, with the long-term involvement of developing nations in mAb production being beneficial to the containment of emerging viral diseases. Given that mAbs have the potential to play a key role in the containment of emerging viral infections, alternative strategies to improve the production of mAbs, including in developing contexts, must be carefully considered.

## Authors’ information

JDJ is a University of Cambridge Wellcome Trust Infection and Immunity PhD candidate.

## Electronic supplementary material

Additional file 1: **Multilingual abstracts in the six official working languages of the United Nations.** (PDF 241 KB)

## Abbreviations

Fab: Fragment antigen-binding
Fc: Fragment crystallisable
EBOV: Ebola virus
mAb: Monoclonal antibody
NHP: Non-human primate
RNA: Ribonucleic acid
scFv: Single-chain fragment variable.
