# Supplementary material for: Leishmania tarentolae: an alternative approach to the production of monoclonal antibodies to treat emerging viral infections
Source: Infect Dis Poverty. 2015 Apr 1;4:8. doi: 10.1186/2049-9957-4-8 (PMC4506428; doi:10.1186/2049-9957-4-8)
Supplement: Supplementary file 1 — Additional file 1: Multilingual abstracts in the six official working languages of the United Nations. (PDF 241 KB) [file 40249_2014_101_MOESM1_ESM.pdf]

Translation of the abstract into the six official working languages of the United Nations

لايشمانية تارينتولا: نهج بديل لإنتاج أجسام الضد وحيدة النسيلة لعلاج حالات العدوى الفيروسية الناشئة

جوشوا د. جونز

الملخـــص

الخلفـــية للعلاج بالأجسام المضادة وحيدة النسيلة دور هام يلعبه وقائيا وعلاجيا بعد التعرض للعدوى الفيروسية، في علاج حالات العدوى الفيروسية التي تشمل حالات العدوى المستجدة. فمثلا، عولج عدة مرضى أصيبوا بفاشية الإيبولا الحالية في غرب أفريقيا بواسطة ZMapp، وهو كوكتيل من ثلاثة أجسام مضادة وحيدة النسيلة معبّر عنها في نبتة *Nicotiana benthamiana*. **نقـــاش** معظم الأجسام المضادة وحيدة النسيلة المستعملة سريريا حاليا معبر عنها في خطوط خلايا الثدييات التي تمنحها طيّا وارتباطا بالغليكوزيل واطنا في الجسم المضاد المعبّر عنه. ويمنح التعبير عن الجسم المضاد وحيد النسيلة في الأجهزة النباتية مزايا تفوق التعبير في خطوط خلايا الثدييات، تتضمن تحسين إمكانية توسيع النطاق وخفض التكاليف. في هذا البحث، أسعى لتسليط الضوء على مزايا نظام أواليّ قادم من أجل التعبير عن أشكال جسم الضد المأشوب. تقدم لايشمانية تارينتولا تعبيرا قويا واقتصاديا عن البروتينات مع نماذج ثديية للارتباط بالغليكوزيل معبّر عنها في خطوط خلوية مستقرة تنمو في معلّق زرع. هناك عدة مزايا لهذا النظام يجعله مناسبا بشكل خاص للاستعمال في أوساط التنمية. **موجـــز** بالنظر إلى الأهمية المحتملة للعلاج بالجسم المضاد وحيد النسيلة في احتواء حالات العدوى الفيروسية المستجدة، يجب استكشاف استراتيجيات مبتكرة وبديلة لتحسين الانتاج.

Translated from English version into Arabic by Lina SM, through

利什曼原虫：生产治疗新发病毒感染的单克隆抗体的另一种方法

Joshua D. Jones

摘要

引言：单克隆抗体在病毒感染（包括新发传染病）治疗中发挥重要作用，主要用于暴露后预防和治疗。例如，目前西非埃博拉病毒疫情中的部分患者所使用的 ZMapp 药物就是本氏烟（*Nicotiana benthamiana*）表达的 3 种单克隆抗体混合物。**讨论：**大部分临床用单克隆抗体由哺乳动物细胞系表达，这些细胞系可提供自然折叠和糖基化的表达性抗体。应用 vegetal 体系表达的单克隆抗体比哺乳动物细胞表达体系更有优势，可提高产量并降低成本。本文重点介绍表达重组抗体的原虫体系的优点。利什曼原虫提供了一个稳健、经济的蛋白表达体系，这些蛋白具有哺乳动物糖基化模型，能在稳定的细胞系中表达，在悬浮培养液生长。这些优点使其更具有发展和应用的价值。**小结：**考虑到单克隆抗体在治疗新发病毒感染方面的潜在重要性，有必要探索创新性和多样性策略提高其产能。

Translated from English version into Chinese by Yin Jian-hai, through

Leishmania tarentolae **: une approche alternative à la production d'anticorps monoclonaux dans le traitement d'infections virales émergentes**

Joshua D. Jones

Résumé

Contexte Le traitement aux anticorps monoclonaux a un important rôle à jouer en tant que prophylaxie et traitement post-exposition dans la prise en charge d'infections virales, y compris d'infections émergentes. De nombreux patients atteints par l'épidémie actuelle du virus Ebola qui sévit en Afrique de l'Ouest ont par exemple été traités au ZMapp, un mélange de trois anticorps monoclonaux exprimés dans une plante de tabac de l'espèce *Nicotiana benthamiana*. **Discussion** La majorité des anticorps monoclonaux faisant l'objet d'un usage clinique sont exprimés dans des lignées cellulaires de mammifères, ce qui permet un repliement et une glycosylation originaux de l'anticorps exprimé. L'expression d'anticorps monoclonaux dans les systèmes végétaux offre des avantages par rapport aux lignées cellulaires de mammifères, y compris une amélioration du potentiel de mise à l'échelle et de réduction des coûts. Je mets en lumière dans cet article les avantages d'un prochain système protozoaire pour l'expression de formats d'anticorps recombinants. *Leishmania tarentolae* permet une expression robuste et économique de protéines présentant des schémas de glycosylation de mammifères dans des lignées cellulaires stables et leur développement dans une culture de suspension. Les nombreux avantages offerts par ce système démontrent qu'il convient particulièrement à une utilisation dans des contextes de développement. **Résumé** Étant donné l'importance potentielle du traitement aux anticorps monoclonaux dans la maîtrise des infections virales émergentes, il convient de développer des stratégies nouvelles et alternatives d'amélioration de la production.

Translated from English version into French by Eric Ragu, through

TRANSLATORS
WITHOUT BORDERS

Leishmania tarentolae**: альтернативный подход к производству моноклональных антител для лечения вирусных инфекций**

Джошуа Д. Джонс

Краткое резюме

Базовая информация Терапия с помощью моноклональных антител играет важную роль как средства постконтактной профилактики и лечения вирусных инфекций, включая развивающиеся инфекции. Например, несколько пациентов, зараженным распространенным на сегодняшний день вирусом «Эбола» в Западной Африке, прошли лечение ZMapp – коктейлем из трех моноклональных антител, выраженных в *Nicotiana benthamiana*. **Обсуждение** Большинство моноклональных антител, применяемых на сегодняшний день для лечения клинических больных, выражены в клеточных линиях млекопитающих, которые обеспечивают исконный обхват и гликозилирование выраженного антитела. Выражение моноклональных антител в вегетативной системе обеспечивает преимущества по сравнению с аналогичным выражением в

клеточных линях млекопитающих, включая повышенный потенциал последующего развития и уменьшение затрат. В настоящей работе я подчеркиваю преимущества формирующейся протозойной системы для выражения форматов рекомбинантных антител. *Leishmania tarentolae* предлагает четкое, экономичное выражение белков со схемами гликозилирования белка млекопитающих, выраженных в устойчивых клеточных линиях и выращенных в суспензионной культуре. Несколько преимуществ данной системы делают особенно подходящей для использования в развивающихся контекстах. **Вывод** Учитывая потенциальную значимость терапии с использованием моноклональных антител в контексте распространяющихся вирусных инфекций, необходимо изучать новые и альтернативные стратегии по улучшению их производства.

Translated from English version into Russian by Irina Zayonchkovskaya, through

Leishmania tarentolae*: un enfoque alternativo a la producción de anticuerpos monoclonales para el tratamiento de infecciones virales incipientes.*

Joshua D. Jones

Resumen

Antecedentes La terapia a base de anticuerpos monoclonales desempeña una función importante como tratamiento profiláctico y terapéutico de infecciones virales, incluidas las infecciones incipientes. Por ejemplo, varios pacientes contagiados en el actual brote del virus del ébola en el occidente de África recibieron tratamiento a base de ZMapp, un coctel de tres anticuerpos monoclonales expresados en la planta *Nicotiana benthamiana*. **Proposición** La mayoría de los anticuerpos monoclonales de uso clínico se encuentran en las líneas celulares de mamíferos, que ofrecen pliegues naturales y la glicosilación del anticuerpo expresado. La expresión de anticuerpos monoclonales en sistemas vegetales ofrece diversas ventajas en cuanto a la expresión en líneas celulares de mamíferos, entre ellas, un mejor potencial de ampliación e intensificación a menor costo. En este documento destaco las ventajas de un sistema de protozoos próximo a desarrollarse para la expresión de formatos de anticuerpos recombinantes. La especie *Leishmania tarentolae* ofrece una expresión de proteínas robusta y económica con patrones de glicosilación en mamíferos expresada en líneas celulares estables creadas en un cultivo de suspensión. Las múltiples ventajas de este sistema lo hacen particularmente apto para usarse en contextos en desarrollo. **Conclusión** Dada la importancia potencial de la terapia a base de anticuerpos monoclonales en el control de infecciones virales incipientes, deben explorarse estrategias alternativas y novedosas para mejorar la producción.

Translated from English version into Spanish by Silvia Sassone, through
